# Mitochondrial ROS production correlates with, but does not directly regulate lifespan in drosophila 

**DOI:** 10.18632/aging.100137

**Published:** 2010-04-15

**Authors:** Alberto Sanz, Daniel J.M. Fernández-Ayala, Rhoda KA Stefanatos, Howard T. Jacobs

**Affiliations:** ^1^ Institute of Medical Technology and Tampere University Hospital, FI-33014 University of Tampere, Finland; ^2^ Present address: Centro Andaluz de Biología del Desarrollo (CABD-CSIC/UPO), Universidad Pablo Olavide, 41013 Seville, Spain

**Keywords:** mtROS, aging, Drosophila, mitochondria, longevity, antioxidants, maximum life span

## Abstract

The Mitochondrial Free Radical Theory of Aging (MFRTA) is currently one of the most widely accepted theories used to explain aging. From MFRTA three basic predictions can be made: long-lived individuals or species should produce fewer mitochondrial Reactive Oxygen Species (mtROS) than short-lived individuals or species; a decrease in mtROS production will increase lifespan; and an increase in mtROS production will decrease lifespan. It is possible to add a further fourth prediction: if ROS is controlling longevity separating these parameters through selection would be impossible. These predictions have been tested in Drosophila melanogaster.  Firstly, we studied levels of mtROS production and lifespan of three wild-type strains of Drosophila, Oregon R, Canton S and Dahomey. Oregon R flies live the longest and produce significantly fewer mtROS than both Canton S and Dahomey. These results are therefore in accordance with the first prediction. A new transgenic Drosophila model expressing the Ciona intestinalis Alternative Oxidase (AOX) was used to test the second prediction.  In fungi and plants, AOX expression regulates both free radical production and lifespan. In Drosophila, AOX expression decreases mtROS production, but does not increase lifespan. This result contradicts the second prediction of MFRTA.  The third prediction was tested in flies mutant for the gene dj-1β. These flies are characterized by an age-associated decline in locomotor function and increased levels of mtROS production. Nevertheless, dj-1β mutant flies do not display decreased lifespan, which again is in contradiction with MFRTA. In our final experiment we utilized flies with DAH mitochondrial DNA in an OR nuclear background, and OR mitochondrial DNA in DAH nuclear background. From this, Mitochondrial DNA does not control free radical production, but it does determine longevity of females independently of mtROS production. In summary, these results do not systematically support the predictions of the MFRTA.  Accordingly, MFRTA should be revised to accommodate these findings.

## Introduction

At present, the Mitochondrial Free Radical Theory of Aging (MFRTA) is one of the most widely believed and supported theories of aging. As well as putting forward an explanation for aging it allows the explanation of inter and intra species differences. According to this theory, free radicals, essentially Reactive Oxygen Species (ROS) which are produced as by-products during normal metabolism inside mitochondria provoke the accumulation of oxidative damage. The accumulation of this oxidative damage is believed to disturb cellular homeostasis which, in turn, is responsible for the aging process. In spite of its attractiveness, MFRTA has received some recent criticism [1,2,3]. Indeed, some evidence indicates that free radicals are part of a complex network of cellular signaling, and not just toxic by-products of metabolism.  Their relationship with aging may therefore be far from straightforward [4].

From MFRTA is it is possible to make three basic predictions: 1) long-lived individuals or species should produce fewer mitochondrial ROS (mtROS) than those which are short-lived, 2) a decrease in mtROS production will increase lifespan and 3) an increase in mtROS production will decrease lifespan. We can add one more prediction: if mtROS is controlling aging then both lifespan and ROS production are inherently linked.   Most evidence in support of MFRTA comes from comparative biology and Dietary restriction (DR) studies, which have attempted to experimentally test the first prediction. It has been shown in several systems that isolated mitochondria from long-lived animals produce fewer mtROS than short-lived ones [5]. It is from these types of studies that a general ‘law' has been proposed, such that lower mtROS production results in a longer lifespan. However, two important exceptions to this law have recently beendescribed [6,7] Ames dwarf mutant mice are the longest-living mouse strain [8], but their mitochondria produce more free radicals than normal controls. Naked-mole rats are the longest-living rodents (Maximum Lifespan (MLS) = 28 years), yet they produce mtROS at the same rate as short-lived mice (MLS= 4 years). Paradoxically, naked-mole rats also have extraordinarily low levels of glutathione peroxidase [9], which could be responsible for the accumulation of unusually elevated levels of oxidative damage in proteins, lipids and nucleic acids [10].

Dietary Restriction (DR) is the only non-genetic treatment that has been shown clearly to increase MLS in most, if not all, species where it has been applied [11]. Since DR decreases mtROS production in isolated mitochondria, a cause and effect relationship has been proposed (reviewed in [12]). However as several different physiological parameters are also coordinately altered during DR, such as insulin signaling [13] and cellular autophagy [14], it is therefore not possible to attribute exclusively this effect on lifespan to simply the attenuation of mtROS production. Moreover, moderate exercise or protein restriction have also been shown to decrease free radical production in a similar way to DR, but do not increase MLS (reviewed in [2]).

In summary, MFRTA is currently mainly supported by indirect data which show a negative correlation between free radical production in isolated mitochondria and lifespan in several different model organisms. However, correlations can suggest but not demonstrate causality. In fact, the only definitive way to test MFRTA is to specifically decrease (or increase) mtROS production and to study the effect of such a modification on lifespan. In the present study we have employed a systematic testing of all basic predictions of MFRTA using such a strategy.

*Drosophila melanogaster* is an excellent model organism to study aging due to its short generation time and lifespan, the availability of the genome sequence and anenormous catalogue of genetic tools. In insects, as in mammals, there is a negative correlation between free radical production in isolated mitochondria and lifespan [15]. Thus, the extreme longevity of queen ants and bees is correlated with a resistance to oxidative stress [16,17]. Evidence from studies in *Drosophila melanogaster*strongly supports MFRTA (reviewed in [18]). For example, oxygen tension modulates *Drosophila* lifespan and gene expression maps are similar in old and chronically hyperoxic flies [19]. Moreover, antioxidant therapies appear to be effective in delaying aging in *Drosophila*  [20,21] , although some authors claim that the increase in lifespan is only produced in short-lived lines [22]  or that it is not related to oxidative damage directly but through the activation of survival-signaling pathways [23]. However, there is also data from *Drosophila *studies that appears to contradict MFRTA. For example, DR has been shown to increase lifespan in *Drosophila *[24] without altering free radical production [25]. It is for these reasons that we have chosen *Drosophila *as model organism to test MFRTA.

We first studied free radical production in three independent wild-type strains of*D. melanogaster* which show a substantial variation in longevity. The results of our study show that the longest-lived strain produces the fewest mtROS which is consistent with MFRTA. However, the longest-lived flies could have characteristics independent of mtROS that might confer the superior longevity. A more rigorous way to examine MFRTA is to test its second prediction by directly manipulating mtROS production. If MFRTA applies, individuals producing fewer mtROS should be long-lived.  Unfortunately, the exact location and mechanism by which free radicals are produced in the electron transport chain (ETC) remains unclear.  This means that any genetic modification to the ETC would most likely result in an increase in free radical production and therefore deleterious effects. However, nature provides some potential solutions to by-pass this problem. Fungi and plants modulate mitochondrial free radical levels through the expression of an enzyme named the Alternative Oxidase (AOX). AOX can by-pass the mitochondrial ETC at complexes III and IV, concomitantly decreasing mtROS generation  [26]. Its expression has been shown to increase lifespan, at least in some fungi [27]. Our group has recently introduced a copy of the AOX gene from the urochordate *Ciona intestinalis *into human cells [28,29] and into *Drosophila melanogaster *[30]. AOX expression confers new physiological properties to cells and animals, such as resistance to ETC inhibitors and partial rescue of metabolic alterations caused by genetic disruption of the ETC complexes or their biosynthesis. We hypothesized that AOX expression would decrease mitochondrial free radical production in* Drosophila* and, if MFRTA is correct, that AOX expression should therefore also increase the lifespan of individuals expressing it. The third prediction was tested using a *Drosophila *mutant, *dj-1β*, which has previously been shown to have increased mitochondrial free radical production in aged flies, manifesting as a severe impairment of locomotive function [30]. If MFRTA is correct, *dj-1β* mutant flies should also be short-lived. Finally, we have selected flies with mitochondrial DNA from the OR long-lived background in DAH short-lived nuclear background (and vice versa), and we have measured both mtROS and lifespan. If MFRTA is correct both parameters should be inherently linked and therefore related such that an alteration of one parameter would translate into a direct effect on the other.

## Results

### Testing prediction #1: "Long-lived individuals should produce fewer mtROS"

In order to test the first prediction we investigated the relationship between levels of mitochondrial ROS production and lifespan in three different wild-type strains of *Drosophila melanogaster *(OR, CS and DAH).

Mitochondrial ROS Production in wild-type strains

**Figure 1.**
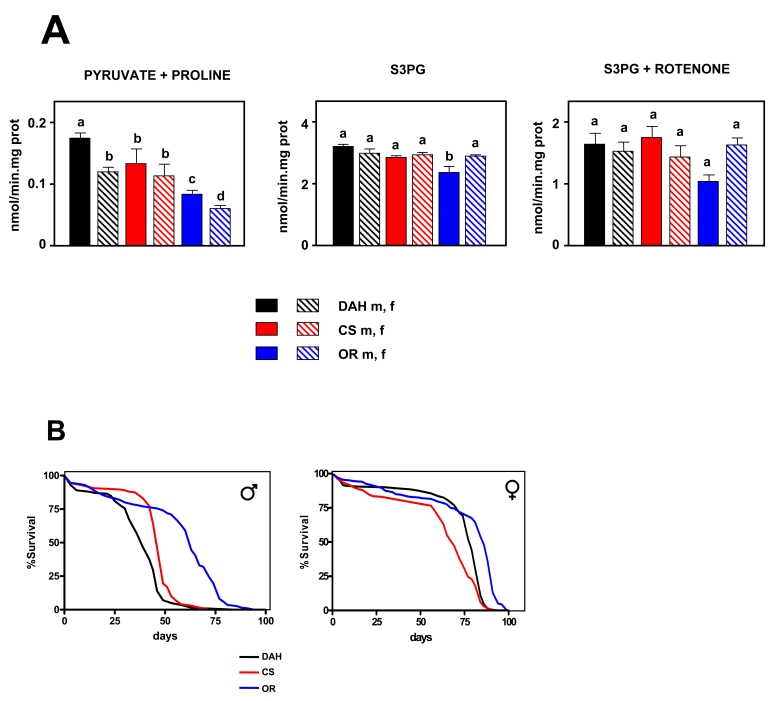
Mitochondrial ROS production *versus* lifespan in three wild type strains of *Drosophila melanogaster*. (**A**) Rate of mtROS production (assayed as H_2_O_2_, mean + SEM). a, b, c and d indicate statistically significant differences between groups (ANOVA, *p* < 0.05, n = 5-9 samples per group),  m: male, f: female.  (**B**) Survival curves.  Combined data from two independent experiments using 100 flies per group per experiment. Mean, maximum lifespans (d) were: DAH males [39,49]; CS males (46, 53); OR males (63, 74); DAH females (79, 84); CS females (69, 81); OR females (86, 91).

mtROS production was measured in 10 day old flies using two different substrates to identify which ETC complex or complexes (if any) are implicated in variation of mtROS  production.  Using a (pyruvate + proline) substrate cocktail, significant differences were detected between groups (*p* < 0.001, Figure 1A). OR flies (both males and females) produced fewer mtROS than the other groups. CS males produced fewer mtROS than DAH males, whereas there were no differences between DAH females and CS flies. OR and DAH females produced significantly fewer mtROS than the corresponding males. Using SP3G as a substrate significant differences were also detected (p < 0.001; Figure 1A), but these were essentially a result of a lower mtROS production of OR males with respect to the other groups. Most of the mtROS production detected using S3PG as substrate is generated during the reverse transfer of electrons between the ubiquinone pool and complex I or by complex III [31]. In relation to aging, complex I seems to be more relevant than complex III (reviewed in [5]).  Therefore, to study in detail the role of complex III in mtROS production, rotenone was added and experiments using S3PG were repeated. When rotenone was present, no significant differences were detected between groups (p = 0.05; Figure 1A). These results indicate that differences between groups are due to variation in ROS produced by complex I, but only when electrons flow in the forward direction. This is in accordance with most published data, supporting an instrumental role of complex I, but not complex III, in mediating variation of mtROS production related to longevity (e.g. [6,34]).

Mitochondrial oxygen consumption

Mitochondrial oxygen consumption was studied in parallel with ROS measurements in order to investigate whether differences in ROS production are related to overall oxygen consumption or to coupling. No differences were detected either in state 4 or state 3 respiration when pyruvate + proline was used as substrate (*p *> 0.05; Table 1). However, RCI was significantly different between the groups (*p*< 0.05), being consistently lower in males than females. Using S3PG (+rotenone) a similar trend was seen (Table 1); no significant differences were found in state 4 or state 3 respiration (*p *> 0.05), but OR males have a lower RCI (*p *< 0.05) males compared to other groups.

 Lifespan studies in wild-type strains

The mean and MLS of females was found to be extended in comparison with males in all the strains studied (Figure 1B). OR males lived 51% longer than DAH males and 40% longer (*p *< 0.001) than CS males. Strikingly, OR males also produced only 53% of the mtROS produced by DAH males and 38% of that produced by CS males when pyruvate + proline was the substrate. OR females lived longer (*p *< 0.001) than DAH and CS females, although differences were much smaller (12% and 8% respectively). Our data are consistent with a direct (inverse) correlation between ROS production at complex I and lifespan in wild-type *Drosophila *strains. In order to confirm such a relationship, we looked at further possible correlations between different parameters associated with ROS generation, oxidative metabolism and MLS. The only significant correlation found was with mtROS production using pyruvate + proline as substrate (Supplementary Figure 1). Interestingly, antioxidants levels analyzed by qPCR (Supplementary Figure 2) negatively correlate with lifespan. This is in agreement with the idea that long-lived strains decrease the generation of damage rather than increase defense or repair in order to increase longevity.

**Table 1.** Mitochondrial oxygen consumption (nmol O _2_/min.mg prot) in three wild type strains of* Drosophila melanogaster*. Results are presented as mean ±SEM. Number of independent samples in parentheses. Different letters (a, b) denote statically significant differences between groups. DAH = Dahomey, CS = Canton S, OR = Oregon R.

	**DAH**		**CS**		**OR**		**ANOVA**
	**males**	**females**	**males**	**females**	**males**	**females**	
	Pyruvate + Proline						
*State 4 *	30 ± 3 (6)	29 ± 4 (6)	28 ± 4 (6)	25 ± 1 (6)	36 ± 3 (6)	23 ± 4 (6)	NS
*State 3 *	313 ± 24 (6)	390 ± 24 (6)	366 ± 16 (6)	393 ± 25 (6)	316 ± 24 (6)	324 ± 24 (6)	NS
*RCI *	10.6 ± 1.1 (6)^a^	15.2 ± 2.1 (6)^b^	13.8 ± 1.1 (6)^a^	15.7 ± 1 (6)^b^	9 ± 1 (6)^a^	14.9 ± 1.7 (6)^b^	*p *< 0.01
	sn-glycerol-3-Phosphate + rotenone						
*State 4 *	77 ± 10 (8)	73 ± 9 (6)	78 ± 10 (8)	70 ± 7 (7)	74 ± 11 (7)	67 ± 6 (8)	NS
*State 3 *	153 ± 19 (8)	187 ± 25 (6)	156 ± 32 (8)	152 ± 19 (7)	116 ± 15 (7)	158 ± 18 (8)	NS
*RCI *	2.2 ± 0.1 (8)^a^	3 ± 0.1 (6)^a^	2.3 ± 0.2 (8)^a^	2.6 ± 0.2 (7)^a^	1.9 ± 0.2(7)^b^	2.6 ± 0.1 (8)^a^	*p* < 0.001

**Table 2.** Mitochondrial oxygen consumption (nmol O _2_/min.mg prot) in in wild type flies (wt) and flies expressing (AOX/da-GAL4), or not expressing AOX (AOX/–). AOX flies are from line F6. For equivalent data for line F24 see Supplementary Table 1.  Results are presented as mean  ±  SEM. Number of independent samples in parentheses.

	**wt**		**AOX/-**		**AOX/da-GAL4**		**ANOVA**
	**males**	**females**	**males**	**females**	**males**	**females**	
	Pyruvate + Proline						
*State 4 *	35 ± 7 (9)	42 ± 9 (10)	32 ± 7 (8)	35 ± 6 (9)	35 ± 5 (7)	46 ± 5 (10)	NS
*State 3 *	410 ± 45 (9)	493 ± 48 (10)	419 ± 41 (8)	495 ± 40 (9)	488 ± 54 (7)	491±35 (10)	NS
*RCI *	16 ± 5 (9)	17 ± 5 (10)	15 ± 3 (8)	19 ± 4 (9)	15 ± 2 (7)	15 ± 1 (10)	NS
	sn-glycerol-3-Phosphate + rotenone						
*State 4 *	65 ± 16 (6)	88 ± 27 (6)	132 ± 28 (8)	112 ± 12 (7)	98± 29 (5)	104 ± 14 (8)	NS
*State 3 *	255 ± 58 (8)	273 ± 35 (6)	254 ± 27 (8)	288 ± 45 (7)	241 ± 26 (5)	276 ± 44 (8)	NS
*RCI *	2.1 ± 0.2 (8)	2.2 ± 0.4 (6)	2.3 ± 0.7 (8)	2.3 ± 0.4 (7)	2.4 ± 0.7(5)	2.9 ± 0.5 (8)	NS

### Testing prediction #2: "A decrease in mtROS production should increase MLS"

Expression of AOX in DAH background

**Figure 2.**
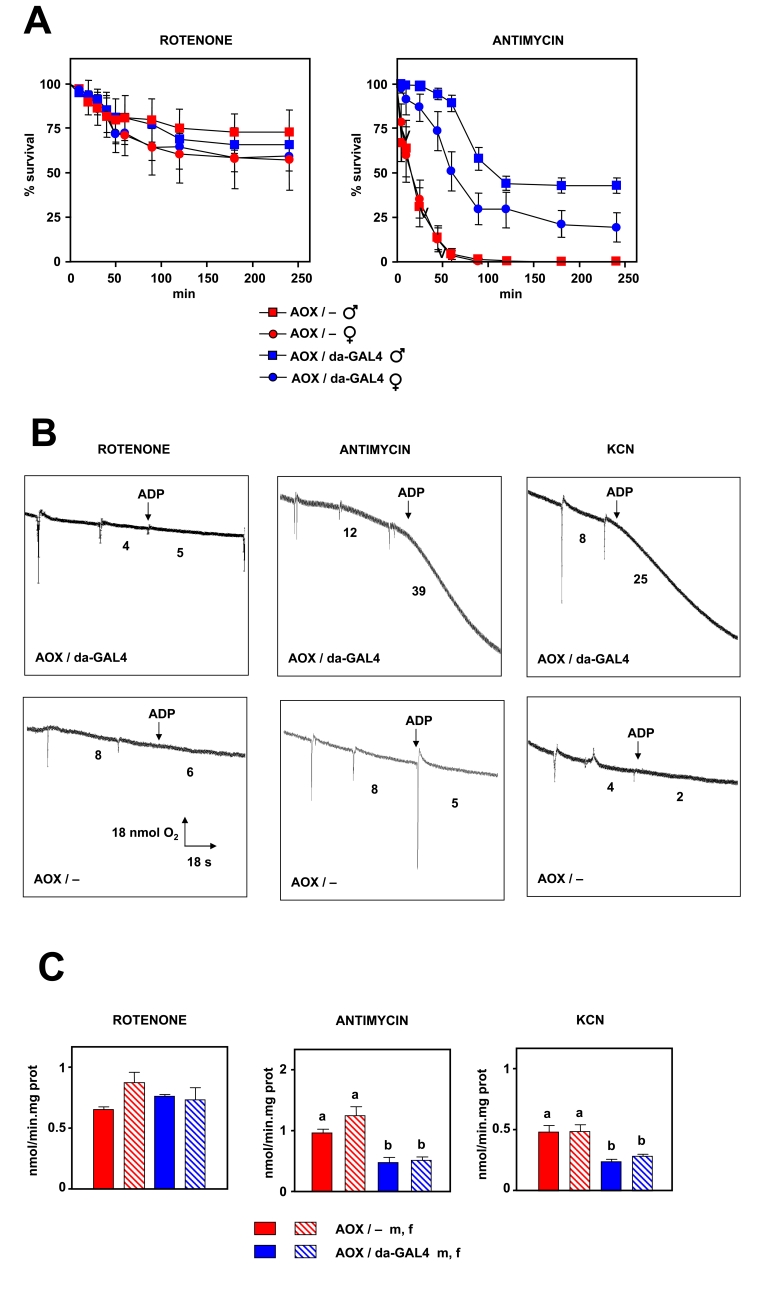
Effects of AOX expression on resistance to respiratory chain inhibitors. (**A**) Survival after exposure to 3 mM rotenone or 3 mM antimycin A, of flies of strains and sexes indicated (AOX / -, flies transgenic for UAS-AOX in absence of GAL4 driver; AOX / da-GAL4, flies transgenic for AOX in presence of da-GAL4 driver). (**B**) Representative oxygraph traces of mitochondrial suspensions (0.5 mg/ml in state 3) in presence of inhibitors shown. Inferred oxygen consumption rates (nmol/min) as indicated. Pyruvate+proline was used as substrate in all experiments. (**C**) mtROS production  (mean + SEM) in presence of inhibitors (at least 4 independent samples per experiment, a, b denote significantly different groups, ANOVA*, p*  < 0.05).

In order to check the second prediction of MFRTA we expressed the alternative oxidase (AOX) of *Ciona intestinalis *in flies, after backcrossing to the DAH background for 11 generations (the flies expressing the *daughterless*-GAL4 driver were also backcrossed in the same conditions).  AOX is able to regulate mtROS generation in plants and fungi, and its expression has been related to an increase in longevity in fungi [26,27]. Firstly, we performed some routine experiments to check the presence and functionality of AOX *in vivo*, in the backcrossed flies. We tested resistance to three different inhibitors of the ETC: 1) rotenone (Complex I), 2) antimycin A (Complex III) and 3) KCN (Complex IV). Flies expressing AOX showed an increased resistance to antimycin A and KCN compared to non-expressing flies (Figure 2A and Supplementary Figure 3). Differences in survival were observed after only 10 min of exposure to drugs inhibiting either complex III or IV. After 24 h of exposure only flies expressing AOX survived.  However, no difference was observed when a complex I inhibitor (rotenone) was employed. In order to confirm that these observations were a result of AOX expression we tested the effect of the inhibitors also on mitochondrial bioenergetics and ROS production. Using isolated mitochondria we observed that AOX is able to support state 3 oxygen consumption in the presence of antimycin or KCN,  but not in the presence of rotenone (Figure 2B). Moreover, AOX decreased mtROS production in the presence of complex III or IV inhibitors, but not in the presence of an inhibitor of complex I (Figure 2C). These data imply that AOX is expressed and is functional *in vivo*. Additionally AOX behaves as theoretically expected, e.g. AOX-expressing flies are resistant to blocks in complex III or IV, but not I.

Effects of AOX on mtROS production and oxygen consumption

**Figure 3.**
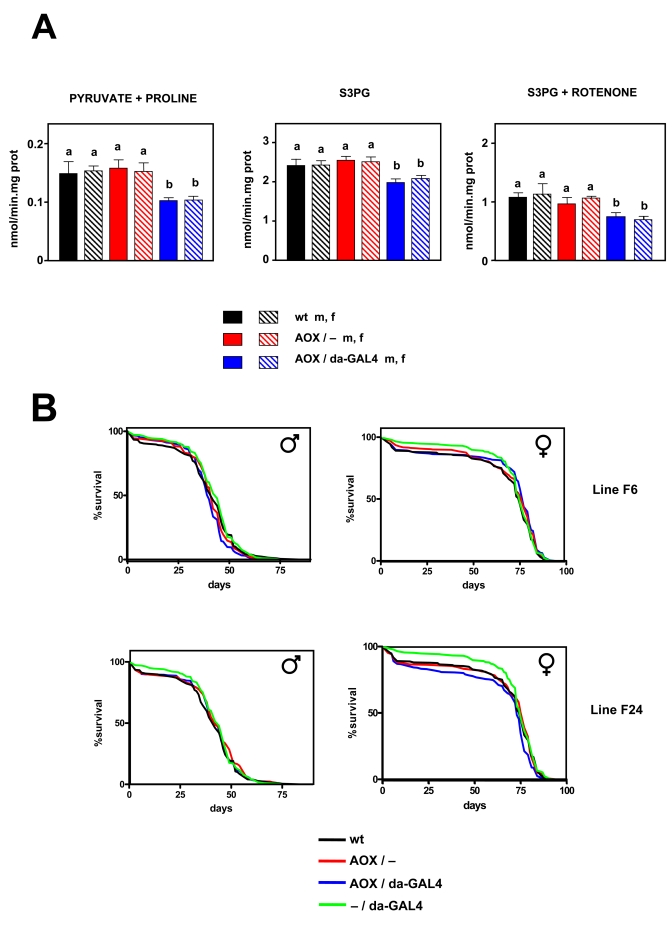
Effect of AOX expression on mtROS production and lifespan. (**A**) mtROS production (mean + SEM). a, b: statistically significant differences between groups (ANOVA, *p* < 0.05, n=4-8 samples per group) m: male, f: female.  (**B**) Survival curves for wild type (wt), AOX non-expressing (AOX / -), AOX expressing (AOX / da-GAL4 +), and driver only (- / da-GAL4) flies, all in the DAH (*w^-^*) background. Flies of AOX transgenic lines F6 and F24 as indicated. Combined data from two independent experiments using 200 flies per group per experiment. Mean, maximum life spans (d) were: wt males (42,51); wt females (75, 82); - / daGAL4 males (44, 54); - / da-GAL4 females (75, 81), F6 AOX / - females (77, 82); F6 AOX / - males (42,51); F6 AOX / da-GAL4 males (40,47); F6 AOX / da-GAL4 females (82, 51); F24 AOX / - males (42, 54); F24 AOX / - females (77, 81); F24 F24 AOX / da-GAL4 males (42, 54); AOX / da-GAL4 females (73, 80).

Having established that AOX was functional *in vivo*, we studied mtROS production in isolated mitochondria in normal conditions (i.e. without inhibitors). The same experiments carried out in wild-type strains were repeated in AOX transgene-expressing and non-expres-sing flies from two independent transgenic lines and in wild type (DAH) controls. AOX was found to decrease mtROS production in 2-3 day old flies when either pyruvate + proline (by 32-34%) or S3PG (by 16-20%) was used as a substrate (Figure 3A, S4). When rotenone was also present in the assay medium AOX flies still produced fewer (27-37%) mtROS than controls with S3PG as substrate (Figure 3A). However; AOX did not modify oxygen consumption in state 3 nor state 4 (Tables 2, S1). We also studied the effects of AOX expression in aged flies. We repeated the same measurements in 30 day old males and 50 day old females, representing equivalent time points in normal male and female lifespan in the DAH background, but under which conditions more than 50% of flies are still alive, thus avoiding the selection of a sub-population.

**Figure 4.**
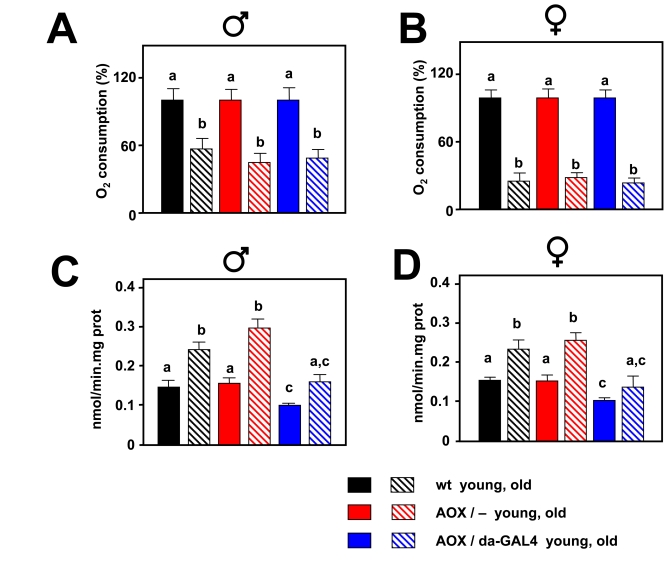
Effect of AOX on mitochondrial bioenergetics and mtROS during aging. Oxygen consumption in state 3 (% of that in the young group) in 30 d old males (**A**) and 50 d old females. (**B)** AOX expression is not able to compensate the decrease in oxygen consumption associated with aging. mtROS generation (nmol H_2_O_2_/min.mg.prot) in 30 d old males (**C**) and 50 d old females (**D**). AOX expression diminishes mtROS production in both young and aged flies, and compensates for the age-associated increase. a, b and c denote statistically significant differences between groups (ANOVA, *p *< 0.05, n = 4-10 samples per group). Pyruvate+proline was used as substrate in all experiments. Plotted data are means ± SEM.

At the ages studied, AOX also decreased mtROS production, both in the presence of KCN and absence of ETC inhibitors (Supplementary Figure 5). During aging mitochondrial oxygen consumption was strongly decreased and AOX was not able to compensate  this  decrease (Figure 4A, B). At the same time mtROS generation was increased, but AOX was able to negate the increase in such a way that mtROS production in old AOX-expressing flies was similar to that in young control flies (Figure 4C, D).

Lifespan and AOX

Lifespan was studied in the same two AOX transgenic- lines (F6 & F24). Both lines have an AOX insertion in an intergenic region, but on different chromosomes (2 and 3, respectively, [30]). Two independent experi-ments each with 200 flies per group were carried out. AOX did not significantly increase lifespan in any of the lines studied (Figure 3B). In males, AOX had a slightly deleterious effect on longevity in line F6 (MLS decreased by around 9%; *p* < 0.05), but none at all in line F24 (*p* > 0.05), whereas in females the opposite was found: no differences were observed in line F6 (*p* > 0.05), but it in line F24 a small decrease (1-3%, *p* < 0.05) was seen. In summary, AOX expression did not consistently or significantly modify lifespan in *Drosophila*.

**Figure 5.**
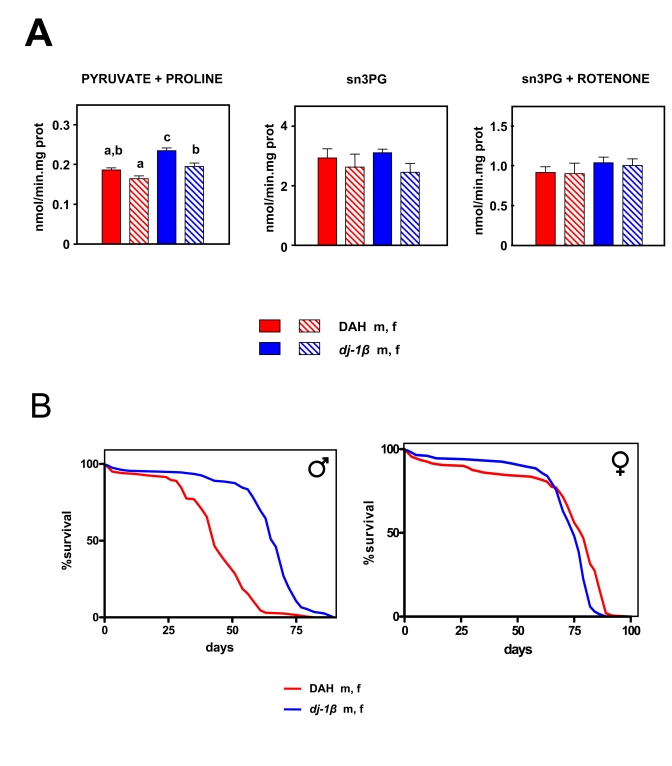
Effects of the dj-1β mutation on mtROS production and lifespan in Drosophila melanogaster. (**A**) mtROS production, assayed as H_2_O_2_, (mean + SEM). a, b and c denote statistically significant differences between groups (ANOVA, p<0.05, n = 4-6 samples per group), m: male, f: female. (**B**) Survival curves.  Combined data from two independent experiments using 100 flies per group per experiment. Mean, maximum lifespans (d) were: DAH males (43, 58); DAH females (75, 79); dj-1β mutant males (67, 75); dj-1β mutant females (79, 86).

### Testing prediction #3: "An increase in mtROS production should decrease MLS"

Mitochondrial free radical production and oxygen consumption in *dj-1β* mutant flies

We measured mitochondrial free radical production in 10 day old *dj-1β* mutant flies using flies from the DAH background as controls. As expected, *dj-1β* mutant flies produced more mtROS than wild-type controls with pyruvate + proline as substrate (Figure 5A; *p* < 0.001), although no significant differences were observed when S3PG was used as a substrate (Figure 5A;* p* > 0.05). Differences in free radical production were not reflected in oxygen consumption (Table 3). Recently, we showed that *dj-1β* mutant flies produce more mtROS than wild-type flies at 3 weeks of age [30].In our previous report only pyruvate + prolinewas used as a substrate. Our present findings confirm these results and clarify the mechanism whereby the *dj-1β* mutation alters mtROS production. Only when electrons flow in the forward direction through complex I are differences detected between mutants and controls. Together, these data support the idea that dj-1β works as a peroxiredoxin [35]. When pyruvate + proline is used as substrate most of the ROS generated are directed to the mitochondrial matrix where *dj-1β* can exert its detoxifying action, whereas when SP3G is used as the substrate ROS production is split between the matrix and the inter-membrane space [31], decreasing the potential role of *dj-1β* in the detoxification process.

Lifespan of *dj-1β* mutant flies

In spite of increased levels of mtROS production *dj-1β* mutant flies were found to have a longer, not shorter lifespan than DAH flies of the corresponding sex: by 30% in males and 9% in females (Figure 5B, *p* < 0.001). Moreover, even after seven generations of backcrossing in to the DAH background (reducing background effects to a minimum) differences in lifespan between mutants and non-mutants for the *dj-1β* were maintained (Supplementary Figure 7A).

**Table 3.** Mitochondrial oxygen consumption (nmol O _2_/min.mg prot) in DAH wild-type and *dj-1β* mutant.

	**DAH**		***dj-1β***	
	**males (6)**	**females (7)**	**males (7)**	**females (10)**
**State 4**	33 ± 5	33 ± 8	38 ± 4	28 ± 5
**State 3**	322 ± 16	396 ± 58	279 ± 37	281 ± 22
**RCI**	13± 2	12±1	9± 1	13 ± 2

Lifespan of *dj-1β* mutant flies

In spite of increased levels of mtROS production *dj-1β* mutant flies were found to have a longer, not shorter lifespan than DAH flies of the corresponding sex: by 30% in males and 9% in females (Figure 5B, *p* < 0.001). Moreover, even after seven generations of backcrossing in to the DAH background (reducing background effects to a minimum) differences in lifespan between mutants and non-mutants for the *dj-1β* were maintained (Supplementary Figure 7A).

### Testing prediction #4: "An increase in mtROS production should decrease MLS"

Analysis of mitochondrial DNA

The nucleotide sequence of mitochondrial gene *coI *was analyzed in three different wild type strains of *Drosophila *melanogaster (OR, DAH and CS) as described in material and methods. We found 10 different polymorphic sites (Supplementary Table 3), although none of the modifications in the gene analyzed caused changes in the amino acid sequence of the protein (all of them were synonymous substitutions). The presence of so many polymorphisms indicates that they could play an important role in *Drosophila *physiology including aging and ROS production and so we investigated the question in more detail.

**Figure 6.**
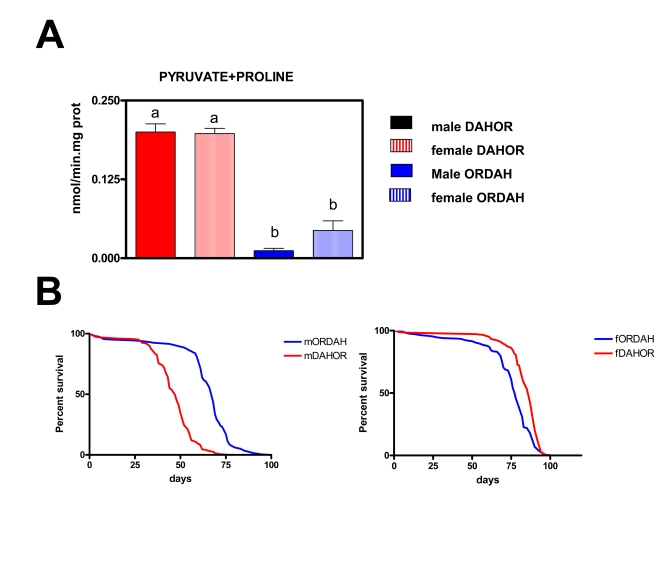
Effects of the changes on mtDNA content on mtROS production and lifespan in new wild type strains of Drosophila melanogaster (DAHOR and ORDAH). (**A**) mtROS production, assayed as H_2_O_2_, (mean + SEM). a and b denote statistically significant differences between groups (ANOVA, p <0.05, n = 4-5 samples per group), m: male, f: female. (**B**) Survival curves.  Combined data from two independent experiments using between 80-100 flies per group per experiment. Mean, maximum lifespans (d) were: ORDAH males (68, 76); ORDAH FEMALES (78, 87); DAHOR males (47,59); DAHOR females (87,92).

ROS production

Initially we wanted to know if mitochondrial ROS production was modulated by polymorphisms in mtDNA so we decided to create new strains of Drosophila melanogaster putting the mitochondrial genome of OR flies in a DAH nuclear background (and vice versa). We measured ROS production in 2/3 days old flies (Figure 6A). At this age we did not find any significant differences between males and females, this mirrors what is seen in the original DAH background where sex differences are only detected after 10 days. ROS production was lower (around 86%) in flies with an OR nuclear background independently of the DAH mitochondrial DNA background. In fact no differences in ROS were found between OR and ORDAH flies or between DAH and DAHOR flies (data not shown).  This data clearly shows that mitochondrial DNA does not control free radical production or at least the free radical production related with longevity in these wild type *Drosophila *strains. Similar results were obtained when OR mtDNA was expressed in a CS nuclear background and vice versa (data not shown), this indicates that the phenomenon is not restricted to the DAH/OR strains.

Life span of DAHOR and ORDAH strains

 Males and females answered differently to changes in the mitochondrial DNA composition. In the males (Figure 6B) the OR background resulted in a longer lifespan (45% mean and 29% MLS) independently of the mitochondrial DNA. This is in agreement with the lower levels of ROS generation in ORDAH flies. However, in females the results were totally opposite, mtDNA determines longevity independently of either nuclear DNA or levels of ROS generation. According to this females with OR mtDNA live longer than females with DAH mtDNA. Mean and maximum lifespan were 12% and 6% (p<0,001) respectively longer in DAHOR females than in ORDAH females in spite of this ROS production was 4.5-fold times higher in the DAHOR females (p<0.001).

## Discussion

The MFRTA is one of themost widely invoked hypotheses accounting for aging, yet the evidence in support of it is almost entirely indirect. In this study we set out to test its predictions experimentally. Although we found a negative correlation between mtROS production and lifespan in 3 wild-type strains of *Drosophila melanogaster *(further support for low level of expression of antioxidants in long-lived individuals) lifespan was not modified as predicted, as a result of genetic manipulations designed to alter mtROS levels. Moreover, we were able to dissociate lifespan and ROS production in wild type strains through changes in the mitochondrial DNA.

A negative correlation between mtROS production in isolated mitochondria and lifespan in flies, mammals and birds [6,15,36] and under conditions of DR [12] has been previously reported. We found a similar relationship, with a long-lived strain (OR) producing fewer mtROS than short-lived strains (DAH and CS), without a major alteration in oxygen consumption. This was more pronounced in males, and held up in females only using a complex I-linked substrate mix.  Our findings thus resolves contradictions of previous studies and emphasizes the importance of studying both sexes and using both complex I- and III-linked substrates. For example, Miwa et al. [25] found no correlation between longevity and mtROS in flies subjected to DR, but their study only looked at females using S3PG as a substrate. Conversely, Sohal et al. [15] did find a correlation using S3PG in males. However, all such correlations provide only indirect support for MFRTA. Interestingly, the expression of 4 antioxidants negatively correlates with lifespan. This supports the idea that long-lived species produce fewer mtROS and consequently need lower levels of antioxidants.

AOX in plants and fungi has been shown to decrease mtROS production when the cytochrome segment of the respiratory chain, but not complex I, is inhibited. When* C. intestinalis *AOX was expressed in *Drosophila *(this study and [30]) mtROS production was similarly diminished in the presence of antimycin or cyanide, but not rotenone.  Furthermore, AOX expression sustained a substantial cyanide- or antimycin-resistant substrate oxidation in mitochondrial suspensions. AOX expression also decreased mtROS production under basal conditions, using either pyruvate + proline or S3PG (with or without rotenone) as a substrate. In plants, the ability of AOX to decrease mtROS production depends on its ability to keep the ubiquinone pool oxidized [37]. The same could apply in *Drosophila*, where semi-ubiquinone at complex I is considered a major site of mtROS generation [31].  We were unable to detect differences in oxygen consumption under basal conditions (i.e. without inhibitors) in AOX-expressing flies, suggesting that AOX does not exert a major effect on respiration *in vivo*. However, it is possible that AOX has a subtle effect on normal respiration that cannot be detected by polarography. The effect on mtROS production of AOX expression in the DAH background was to diminish it to levels similar to those of wild-type OR flies. In addition, AOX suppressed the age-associated increase in mtROS production, but not the age-associated decrease in substrate oxidation by isolated mitochondria [38,39]. Since AOX expression produced no significant effect on lifespan, this is consistent with the idea that a mitochondrial parameter other than ROS production could be a determinant of aging, as proposed e.g. by Trifunovic and Larsson [40]. Our data showing that mtDNA composition could regulate lifespan supports such an idea.

Previous attempts to test the MFRTA by disruption of complex II, both in *Drosophila  *[41] and *Caenorhabditis *[42] are limited by the fact that this treatment clearly produces pleiotropic effects on energy metabolism and development. In contrast, the *dj-1β* mutation produces no negative effect on development or fecundity, its only known phenotypes being age-associated loss of locomotor function and a hypersensitivity to paraquat.  Both of these have been attributed to the deficit of mitochondrial antioxidant capacity, as manifested by increased mtROS production compared with that of wild-type strains, which we confirmed *in vitro*, using flies of different ages. Indeed, the locomotor deficiency is corrected by AOX expression [30], which correlates with decreased mtROS production.  Nevertheless, the *dj-1β* mutation did not result in shortened lifespan.  Surprisingly, the opposite was observed, with the lifespan *dj-1β* flies comparable with that of wild-type OR flies*. *Moreover, even after backcrossing the *dj-1β* mutants for seven generations into a DAH background differences in longevity were still present. Surprising, the level of expression of antioxidants in dj-1β mutants is reduced when compared to controls. This indicates that compensation in antioxidant levels can not account for the long lifespan of *dj-1β* mutants. Even more surprising is the fact that correlation between mtROS and lifespan is lost when *dj-1β* flies are included, but the correlation between antioxidant levels and lifespan becomes more significant when *dj-1β* are included in the correlation (Supplementary Figure 8B,C). DAH background was used as a control for *dj-1β* (in experiments using either isogenic or non-isogenic lines) in order to keep the flies in experiments increasing (*dj-1b mutants*) or decreasing (AOX) ROS the same. For this reason, it may be argued that a decrease in lifespan may be found when *dj-1β* mutation is expressed in a long-lived background (e.g. OR). In any case,  ROS themselves are bad predictors of lifespan as both OR and *dj-1β* are long-lived (compared to DAH) in spite of opposite levels of ROS.

One possible objection to our conclusion that AOX does not increase lifespan despite diminishing mtROS production would be that the enzyme may not be functional under normal physiological conditions.  However, its ability to complement several mutations affecting cytochrome oxidase function, as well as the toxicity of cyanide and antimycin *in vivo* and the overproduction of ROS*in vitro* and *in vivo* caused by the *dj-1β* mutation [30] suggests otherwise.  It can also be argued that an "exogenous" protein cannot increase the lifespan of the host organism. However, it has been previously shown that the expression of human UCP-2 increases *Drosophila *lifespan. And moreover, we and others have recently demonstrated that it is possible to increase *Drosophila *lifespan by expressing: NDI1 that as AOX is not encoded in the animal host genome [43,44]. Interestingly, the expression of NDI1 (a protein that can by-pass mitochondrial complex I) significantly increases lifespan without decreasing the basal rate of ROS production.

We also cannot exclude that AOX has other, undetected effects influencing lifespan, which over-ride those mediated through decreased mtROS production. Note, however, that AOX expression has only a minimal effect on the development or physiology of wild-type flies [30] and it does not alter the expression of major antioxidants (Supplementary Figure 6). Certainly, we cannot discard either that the expression of AOX in certain tissues (e.g. the nervous system) or during different life stages (e.g. in the last part of life) may have a different effect on longevity. A similar point could be made with regard to *dj-1β* mutant flies, i.e. that increased mtROS production *in vitro* and paraquat sensitivity *in vivo *do not reflect a systematic effect on mtROS levels *in vivo *under normal physiological conditions. Thus, the over-production of ROS in *dj-1β*mutant flies could be compensated by the alteration of another function of the protein. DJ-1 participates in RNA metabolism and transcription [45] so its effects on gene expression could compensate for the over-generation of ROS. However, all these caveats do not apply to our fourth experimental approach. Where mitochondrial DNA of OR is expressed in a DAH nuclear background (and vice versa) ROS production is not altered (it is totally determined by the nuclear background), but lifespan of females is significantly changed depending on the mitochondrial background. The fact that DAHOR females are a long-lived strain in spite of high levels of mtROS production is a strong argument against MFRTA. Moreover, this demonstrates that it is possible to separate lifespan and ROS production in wild type strains of *Drosophila.*

*Drosophila melanogaster *strains selected for long or short lifespan [46] exhibit differences in several physiological parameters (including mtROS production and the levels of antioxidant proteins).  It has been suggested that longevity evolves through coordinated changes in multiple genes and biochemical pathways [47], which could accommodate our results by postulating that altered mtROS production or detoxification cannot have a material effect on lifespan without concomitant changes in other pathways, such as protein acetylation, insulin signaling or alterations in the degree of un-saturation of lipids in biological membranes.  Moreover, a lifespan-increasing effect in one parameter, such as a decrease in mtROS production, could result in a compensatory change in another, such as the repair proteins of DNA, resulting in no net alteration in lifespan. Once time more the results of DAHOR and ORDAH females support such hypothesis.

Regardless of the molecular reasons, our findings indicate that mtROS production is not and cannot be the sole determinant of lifespan in *Drosophila*, strengthening similar conclusions arrived at recently in studies of naked mole rats [9,10], long-lived *Ames dwarf* mice [7] and *C. elegans  * [48]. However, our findings are subject to two important caveats. First, the use of in vitro assays to measure mtROS production, which may not reflect the situation in vivo. And second, the assumption that AOX expression or the mutational downregulation of *dj-1β* does not produce pleiotropic or off-target effects that negate or over-ride effects on mtROS (see above).

The first caveat is one shared with the great majority (if not all) of studies supporting MFRTA. In fact, it is still not known if long-lived animals produce fewer free radicals than short-lived ones in vivo. Further, it has only been demonstrated that isolated mitochondria from long-lived animals produce fewer molecules of H_2_O_2_(data about superoxide are contradictory). Additionally, differences in ROS generation in isolated mitochondria are only observable under certain experimental conditions. For example, it is currently assumed that dietary restricted animals produce fewer mtROS than ad libitum-fed animals. However, differences are only observed when pyruvate (+malate) (or glutamate (+malate)) are used as a substrate [12]. On the other hand, the use of isolated mitochondria is required due to the lack of a sensitive method that would allow quantification of specific free radical species in cells (reviewed in [49]). As an example of this type of situation we could mention the work carried out in Brian Merry's laboratory. His laboratory had previously reported differences in ROS production between ad libitum and caloric restricted rats using isolated liver mitochondria [50]. However, no differences were observed in the same experimental model when intact hepatocytes were used for these measurements [51].

 In summary, in order to test MFRTA we chose an original strategy of trying to modulate the generation of damage and not just increase antioxidant defense or repair mechanisms. With this we avoid the drawbacks and caveats of the latter approach [5]. In fact, even if our results do not reflect the situation in vivo, they are enormously relevant since they clearly dissociate -for the first time- levels of mtROS production in isolated mitochondria and longevity in *Drosophila melanogaster*, indicating that other mitochondrial factors such as the presence of polymorphisms in mitochondrial DNA may act as longevity regulators.

## Materials and methods

Flies. *Drosophila *wild-type strains Dahomey (DAH), Canton S (CS) and Oregon R (OR) were obtained from stock-centers or collaborators. The *dj-1β^GE23381^* mutant [31]: and AOX-transgenic lines F6 and F24 [30] were as described previously. Flies were maintained in a standard medium [30], collected using CO_2  _anesthesia within 24 h of eclosion, and then kept at a density of 20 flies per vial at 25 ºC in a controlled 12 h light:-dark cycle. Vials were changed every 2-3 days. We have created two new wild type strains of *Drosophilamelanogaster* backcrossing for eleven generations DAH virgin females with OR males and OR virgin females with DAH males. The new strains of *Drosophila melanogaster *are called DAHOR (flies with nuclear DAH DNA and mitochondrial OR DNA) and ORDAH (flies with nuclear OR DNA and mitochondrial DAH DNA).

Lifespan studies. Between 180 and 400 flies were used for each study. Each independent study was repeated twice: data were pooled and analysed together. Flies were collected within 24 h after eclosion using CO_2 _anaesthesia and kept at a density of 20 flies per vial at 25 ºC in a controlled 12 h light:-dark cycle. Every 2-3 days vials were changed and the number of dead flies was counted, from which mean and maximum lifespan (MLS, the last 10% of surviving flies) were calculated. Prism GraphPad software was utilized to build survival curves that were further analysed using the Kaplan Meier Log-Rank Test.

Mitochondrial biochemistry. Mitochondria were isolated according to Miwa et al.[31] with some minor modifications [30]. Mitochondrial respiration rates were measured by polarography using a Clark-type oxygen electrode as previously [30], in the absence or presence of KCN (100 μM), antimycin A (10 μM) or rotenone (5 μM). Mitochondrial ROS production was assayed according to the method described by [32] adapted to flies [30].

RNA quantification. Total RNA was extracted from 10 days old flies according to [30]. For cDNA synthesis, 13 μl reaction mixes containing 2 μg RNA, 1 μl DEPC 10 mM dNTP mix (Fermentas), 0.4μl Random Primers (0.5ug/μl Promega) and DEPC-treated water were incubated at 90°C for 3 min, then transferred to ice, where 4 μl 5x M-MuLV reaction buffer (Fermentas) and 1μl  40U/μl RNase inhibitor (Fermentas) were added. The reactions were mixed and incubated at 25°C for 10 min. On ice, 2μl of 20U/μl M-MuLV reverse transcriptase (Fermentas) was added, and the reaction was incubated for a further 10 min at 25°C, 1 h at 37°C and 70°C for 10 min. mRNA levels were analyzed by Q-RT-PCR. The transcript levels of *RpL32, Catalase, Superoxide dismutase 1 and 2 and Glutathione Peroxidase *were measured using primers pairs shown in supplementary Table 2. All RNA extractions were performed in triplicate, with each used as a template for three separate cDNA synthesis reactions which were then pooled. Each cDNA pool was itself analysed in triplicate. Expression of the target genes was measured relative to that of *RpL32 *(rp49), in order to normalize for sample and run to run variations. A series of 10-fold dilutions of an external standard was used in each run to produce a standard curve. Analytical reactions were performed using 20-fold diluted cDNA samples, in 25 μl reaction volume consisting of 2 μl of the cDNA template, 0.4μl of 20 μM forward and reverse primers, and 12.5ul of 2x MAXIMA SYBR GREEN Master Mix (Fermentas). The PCR program consisted of a 10 min pre-incubation at 95°C, 40 cycles of 35 secs denaturation at 95°C, 30 secs annealing at 60°C and 30 secs extension at 72°C.  Melting curve analysis, consisting of a 15 secs  denaturation step at 95°c followed by a 1 min annealing step at 60°C and a 0.3°C/s denaturation ramp to 95 °C, was performed after the amplification step to verify that only a single, specific extension product had been amplified. Data were extracted and analysed using Applied Biosystems StepOne software version 2.0.

Resistance to inhibitors of the ETC. To check the expression and activity of AOX *in vivo* experiments with a variety of ETC inhibitors were performed.  20 flies were kept (males and females separately) in fresh vials. To measure resistance to KCN, the drug was dissolved in water at varying concentrations and added directly to the food vial. Resistance to antimycin and rotenone was assayed essential as described by Fridell et al. [33]. In brief, 2-3 day old flies were starved for two hours in empty vials, following this flies were placed in vials containing Whatman paper (3 mm x 1 mm) impreg-nated with 5% (w/v) sucrose solution and the appropriate drug (3 mM antimycin or rotenone). Under these conditions without any drug, flies are able to survive more than 72 h so any effect before this time should be considered to be provoked by exposure to the drug. The proportion of flies surviving was recorded over 24 h.

Sequencing of Mitochondrial gene cytochrome c oxidase subunit I. Mitochondrial DNA was extracted using standard procedures from mitochondria isolated from around 150 flies according to *Miwa et al*. [31] High fidelity PCR using specific primers CoIF2 and CoIR5 (Supplementary Table 2)  were used to amplify a 2.6 kb fragment containing the cytochrome c oxidase subunit I (CG34067, CoI).  PCR products were purified using a Machary - Nagel PCR purification kit according to manufacturer's instructions.  Products were sequenced using Big dye Terminator Chemistry 3.1v (Applied Biosystems) and a 3130 AB genetic analyser. AB sequencing analysis software was used for analysis of electropherograms.

Statistical analysis. Data were analysed using GraphPad Prism 4 and one-way ANOVA was used for statistical testing. When ANOVA was significant (*p *< 0.05) Newman-Keuls Multiple Comparison test was also used. Lifespan data were analysed using the Kaplan Meier Log-Rank Test.  The statistically significant value was established as *p* < 0.05.

## Acknowledgments

Our work is supported by funding from the Academy of Finland, Tampere Hospital Medical Research Fund, Juselius Foundation, the European Union and  EMBO (long-term fellowship to AS). We thank Dr. J Chung for supplying the dj-1beta mutant stock.

## Supplementary data

Supplementary Figure 1 Correlation between mtROS production, oxygen consumption and maximum life span in three wild-type strains of *Drosophila melanogaster*. The statistical relationships between MLS and various parameters related to mitochondrial function were analyzed using linear regression (equation y = a + bx). Only mtROS production (using pyruvate + proline as substrate) was significantly correlated with lifespan.

Supplementary Figure 2 Level of expression of four antioxidant genes in three wild-type strains of *Drosophila melanogaster*. (**A**) The level of expression of four antioxidant genes: catalase (CAT), glutathione pero-xidase (PHGPx), superoxide dismutase 1 (SOD1) and superoxide dismutase 2 (SOD2) were analyzed by qPCR. Plotted data are mean + SEM. a, b and c denote statistically significant differences between groups (ANOVA, p < 0.05, n = 6 samples per group) (**B**) The statistical relationships between MLS and the expression of CAT, PHGPx, SOD1 and SOD2 were analyzed using linear regression (equation y=a + bx) . All the antioxidants show a negative correlation with MLS, but only in the case of SOD2 the correlation is statistically significant (p<0.05).

Supplementary Figure 3 Survival of AOX-expressing and non-expressing flies 24 h after acute exposure to KCN. High doses of KCN were lethal to all non-expressing (AOX/–) flies, but some AOX-expressing (AOX/da-GAL4) flies survived, whereas low doses had no effect on survival of any group. 80 flies were used per group and per experiment. Asterisks denote significant differences between groups at the same dose level (ANOVA, p<0.05).

Supplementary Figure 4 Mitochondrial ROS production in AOX expressing and non-expressing flies from transgenic line F24. Genotypes as in Fig. 3.  a, b  denote statistically significant differences (ANOVA, p<0.05, n = 4-6 per group). Plotted data are mean rates of H2O2 production ? SEM.  m = male; f = female.

Supplementary Figure 5 Mitochondrial ROS production in aged wild-type (wt), AOX expressing and non-expressing flies. AOX flies from transgenic line F6, genotypes as in Figure 3.  Pyruvate + proline in the presence or absence of KCN was used as substrate. a, b denote statistically significant differences (ANOVA, p<0.05, n = 5-6 samples per group). Males were 30 d old and females 50 d old. Plotted data are mean rates of H2O2 production ± SEM.

Supplementary Figure 6 Level of expression of four antioxidant genes in fliex expressing and non-expressing AOX. (**A**) The level of expression of four antioxidant genes: catalase (CAT), glutathione peroxidase (PHGPx), superoxide dismutase 1 (SOD1) and superoxide dismutase 2 (SOD2) were analyzed by qPCR. Plotted data are mean + SEM. a, b and c denote statistically significant differences between groups (ANOVA, p < 0.05, n =5-6 samples per group).

Supplementary Figure 7 Life span and level of expression of four antioxidant in dj-1β mutant flies. (**A**) Survival curves of dj-1β  mutant flies backcrossed during seven generation in dahomey background. Combined data from two independent experiments using 100 flies per group per experiment Mean, maximum lifespans (d) were: DAH males (46, 55); DAH females (71, 76); dj-1β mutant males (55, 64); dj-1β mutant females (85, 88). (**B**) The level of expression of four antioxidant genes: catalase (CAT), glutathione peroxidase (PHGPx), superoxide dismutase 1 (SOD1) and superoxide dismutase 2 (SOD2) were analyzed by qPCR. Plotted data are mean + SEM. * denotes statistically significant differences between groups (T-test, p<0.05, n =5-6 samples per group).

Supplementary Figure 8

Supplementary Table 1 Mitochondrial oxygen consumption (nmol O2/min.mg prot) in AOX-expressing (AOX/da-GAL4) and non-expressing (AOX/–) transgenic flies. AOX transgenic flies were from line F24 and were 2-3 d old. Results are mean ± SEM. Number of independent samples in parentheses

Supplementary Table 2 Primer sequences used for qPCR and sequencing.

Supplementary Table 3 Nucleotide variation at polymorphic sites of the cytochrome c oxidase I (CoI) gene in three wild type strains of *Drosophila melanogaster*.
