# Supplementary figures and images for: Mitochondrial ROS production correlates with, but does not directly regulate lifespan in drosophila
Source: Aging (Albany NY). 2010 Apr 15;2(4):200–23. doi: 10.18632/aging.100137 (PMC2880708; doi:10.18632/aging.100137)

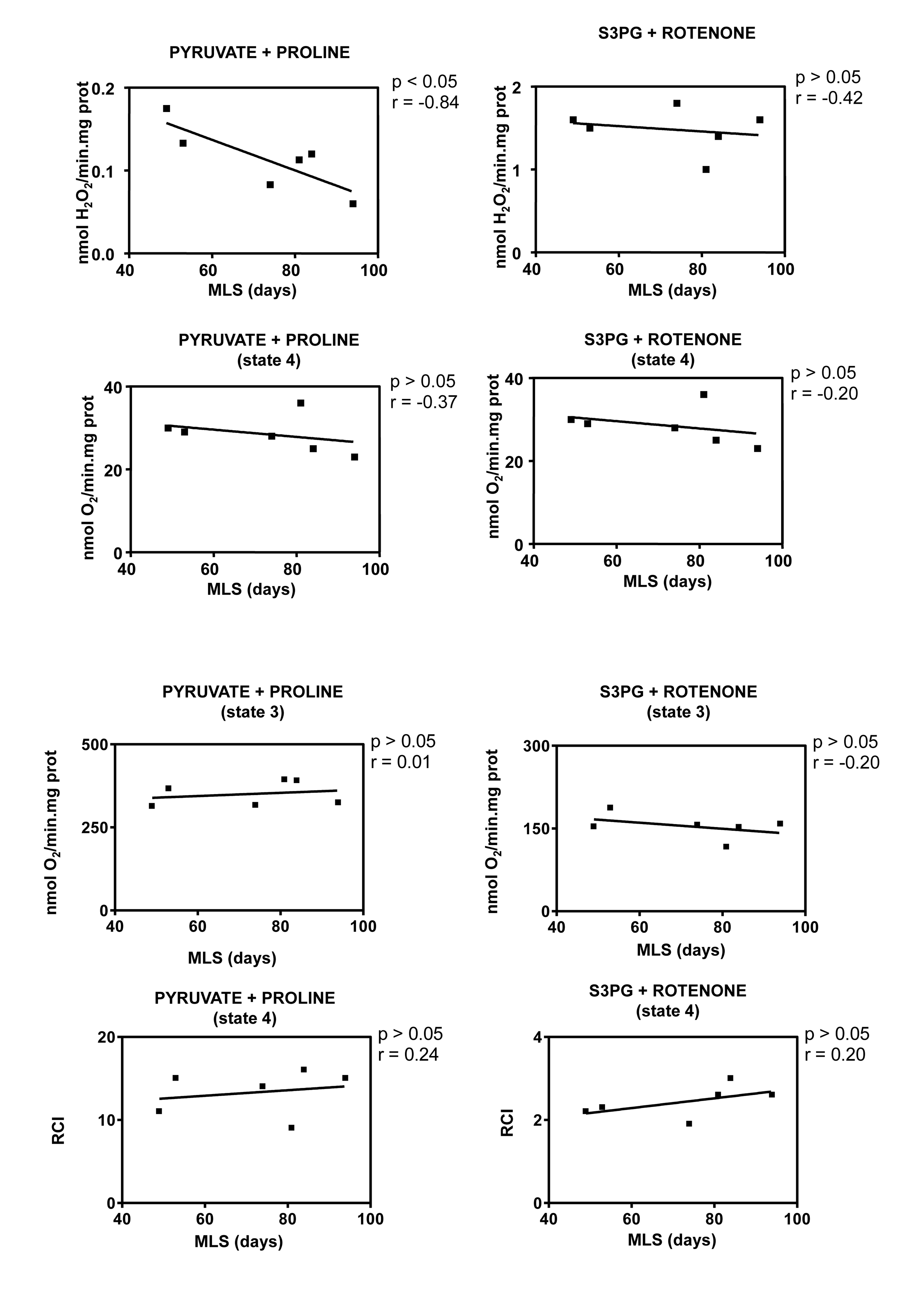

Supplement: Supplementary Figure 1 — The statistical relationships between MLS and various parameters related to mitochondrial function were analyzed using linear regression (equation y = a + bx). Only mtROS production (using pyruvate + proline as substrate) was significantly correlated with lifespan. [file aging-02-200-s001.tif]

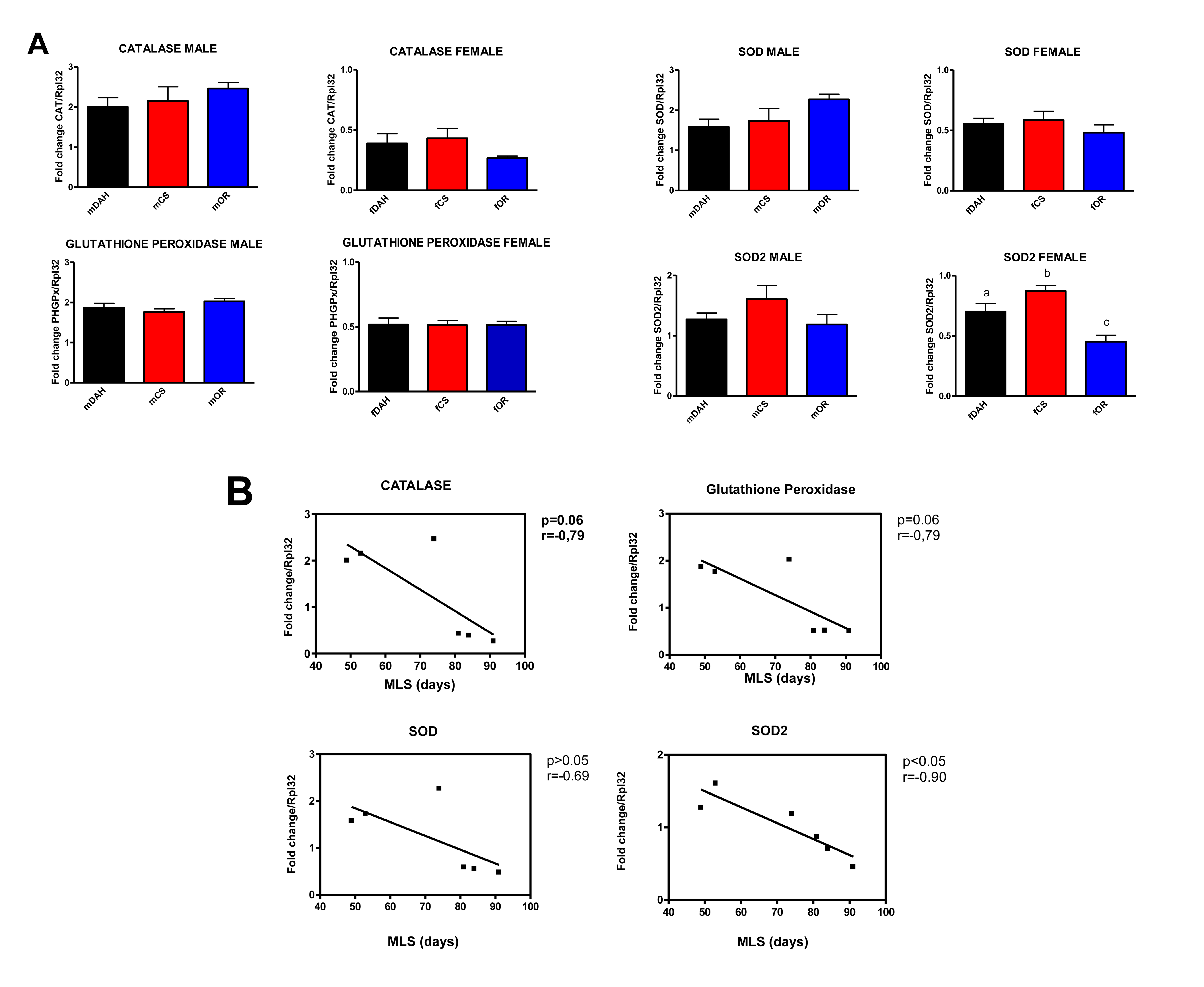

Supplement: Supplementary Figure 2 — (A) The level of expression of four antioxidant genes: catalase (CAT), glutathione pero-xidase (PHGPx), superoxide dismutase 1 (SOD1) and superoxide dismutase 2 (SOD2) were analyzed by qPCR. Plotted data are mean + SEM. a, b and c denote statistically significant differences between groups (ANOVA, p < 0.05, n = 6 samples per group) (B) The statistical relationships between MLS and the expression of CAT, PHGPx, SOD1 and SOD2 were analyzed using linear regression (equation y=a + bx) . All the antioxidants show a negative correlation with MLS, but only in the case of SOD2 the correlation is statistically significant (p<0.05). [file aging-02-200-s002.tif]

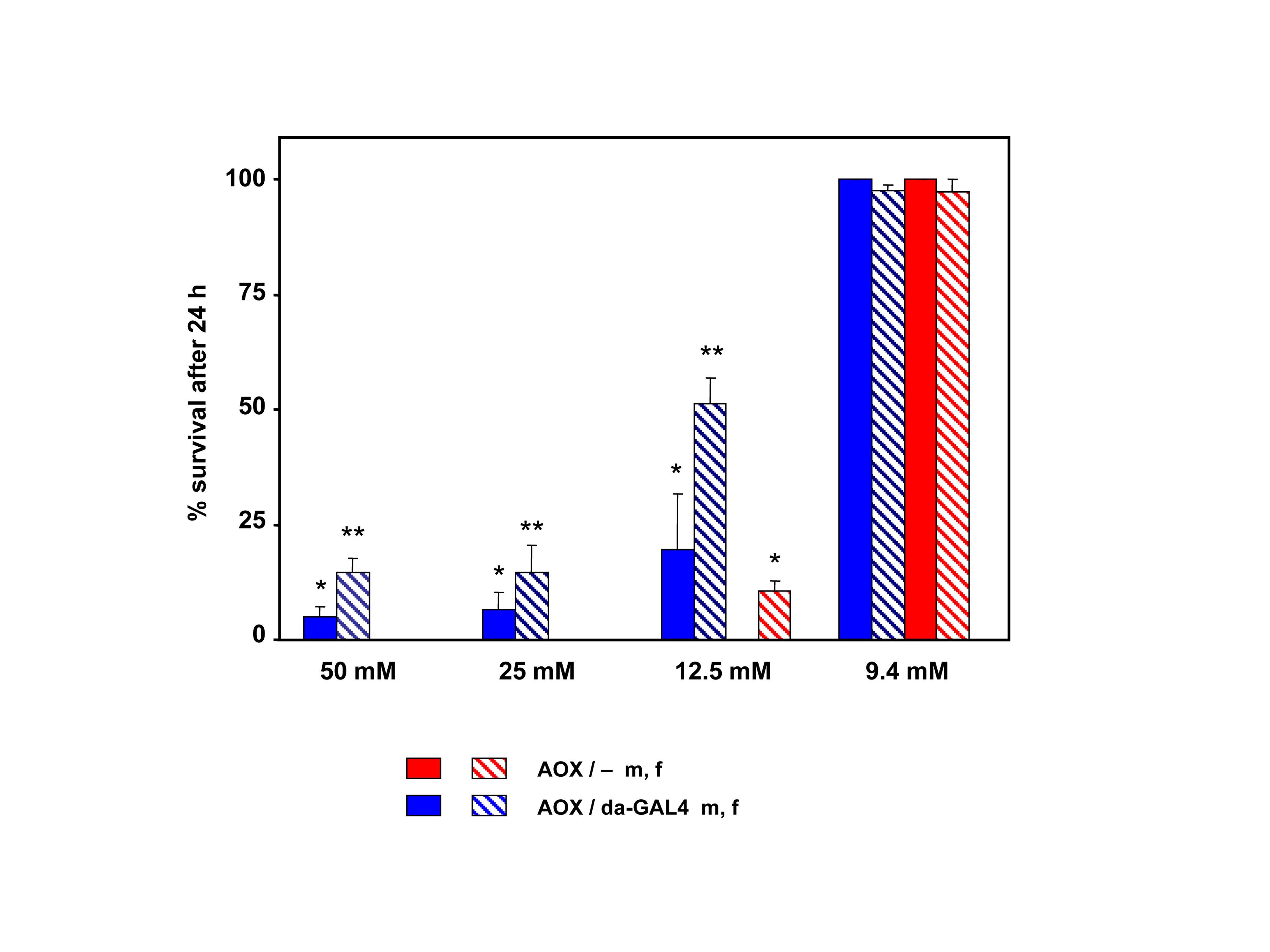

Supplement: Supplementary Figure 3 — High doses of KCN were lethal to all non-expressing (AOX/–) flies, but some AOX-expressing (AOX/da-GAL4) flies survived, whereas low doses had no effect on survival of any group. 80 flies were used per group and per experiment. Asterisks denote significant differences between groups at the same dose level (ANOVA, p<0.05). [file aging-02-200-s003.tif]

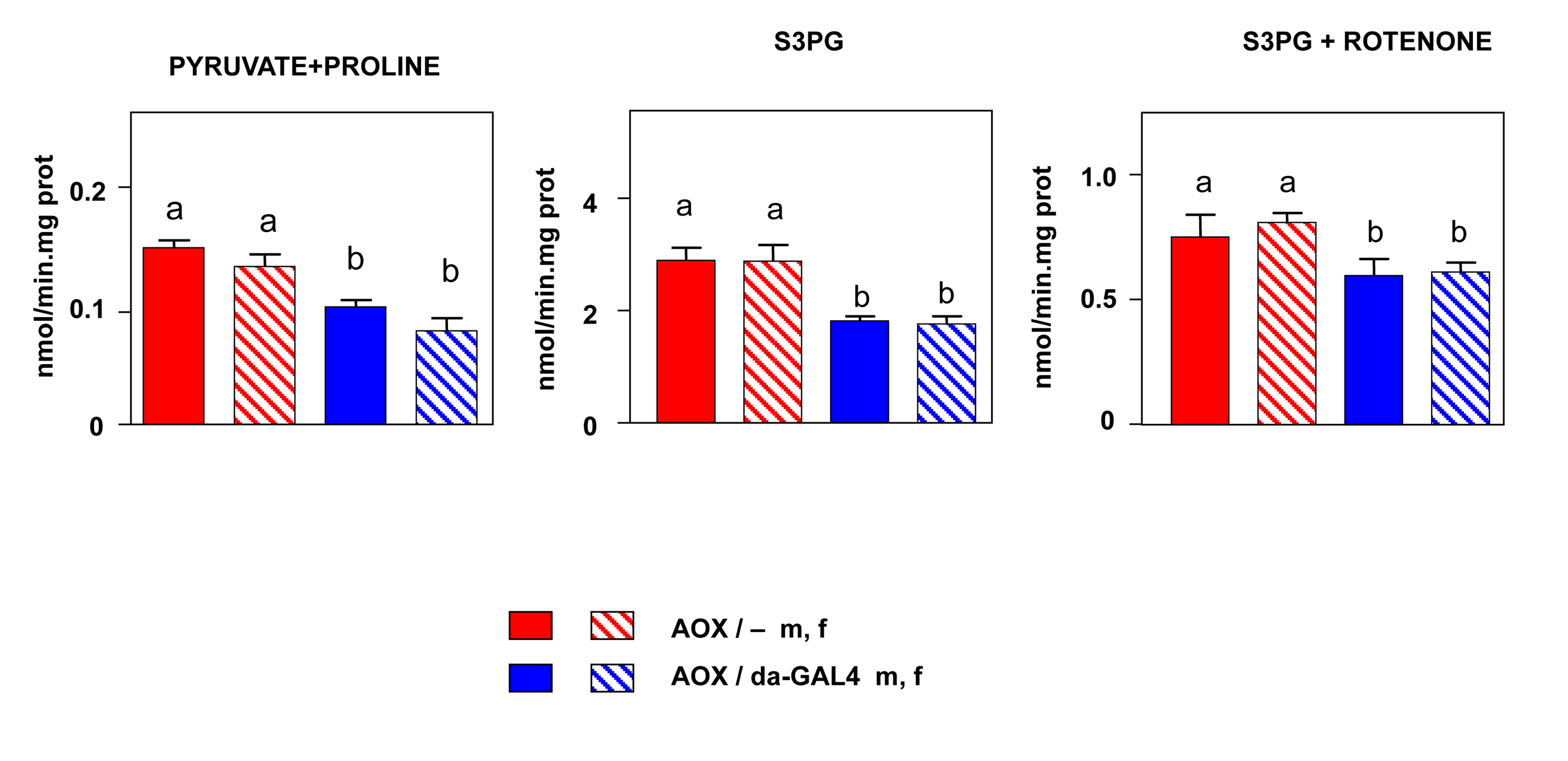

Supplement: Supplementary Figure 4 — Genotypes as in Fig. 3. a, b denote statistically significant differences (ANOVA, p<0.05, n = 4-6 per group). Plotted data are mean rates of H2O2 production ? SEM. m = male; f = female. [file aging-02-200-s004.tif]

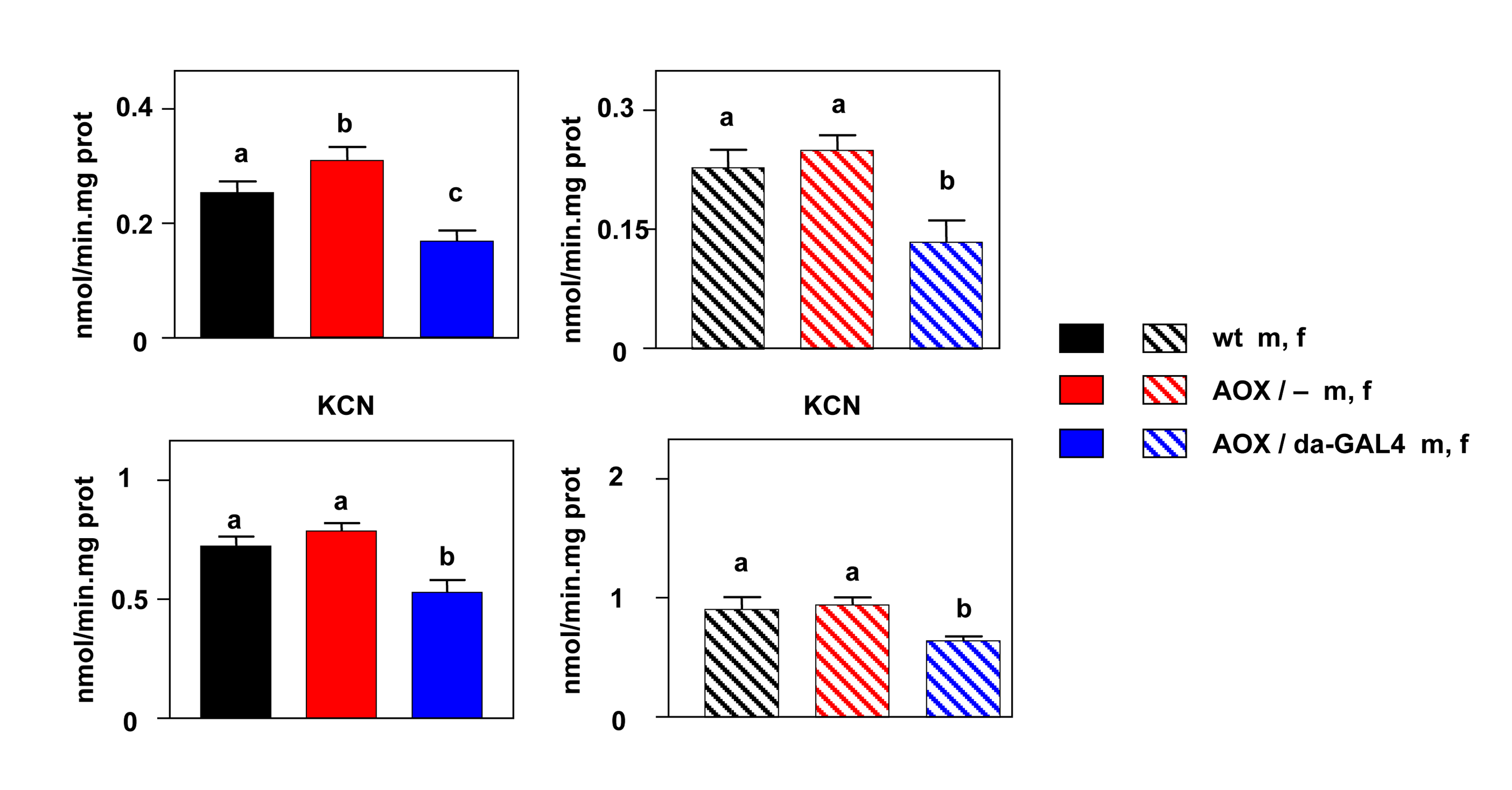

Supplement: Supplementary Figure 5 — AOX flies from transgenic line F6, genotypes as in Figure 3. Pyruvate + proline in the presence or absence of KCN was used as substrate. a, b denote statistically significant differences (ANOVA, p<0.05, n = 5-6 samples per group). Males were 30 d old and females 50 d old. Plotted data are mean rates of H2O2 production ± SEM. [file aging-02-200-s005.tif]

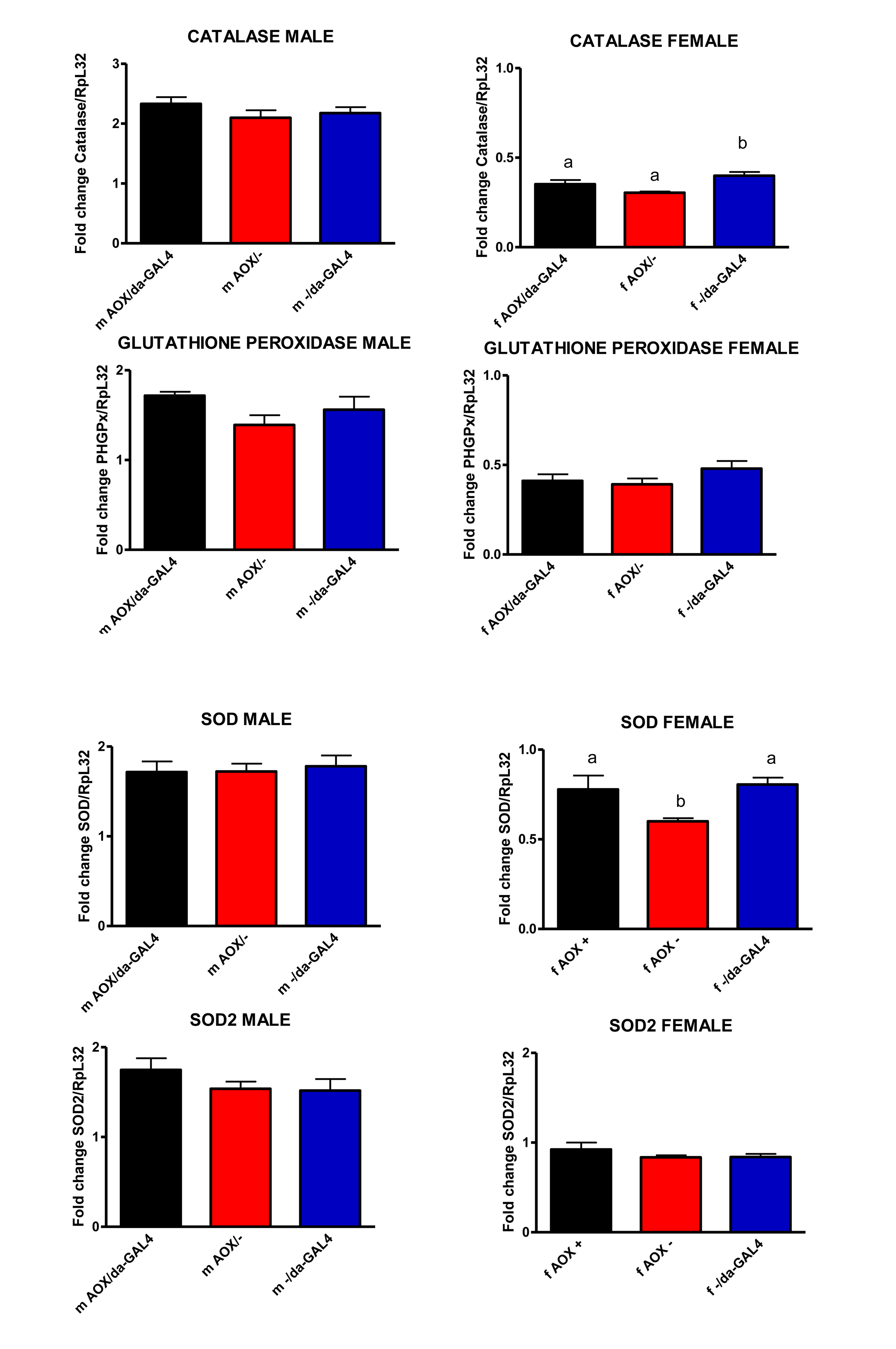

Supplement: Supplementary Figure 6 — (A) The level of expression of four antioxidant genes: catalase (CAT), glutathione peroxidase (PHGPx), superoxide dismutase 1 (SOD1) and superoxide dismutase 2 (SOD2) were analyzed by qPCR. Plotted data are mean + SEM. a, b and c denote statistically significant differences between groups (ANOVA, p < 0.05, n =5-6 samples per group). [file aging-02-200-s006.tif]

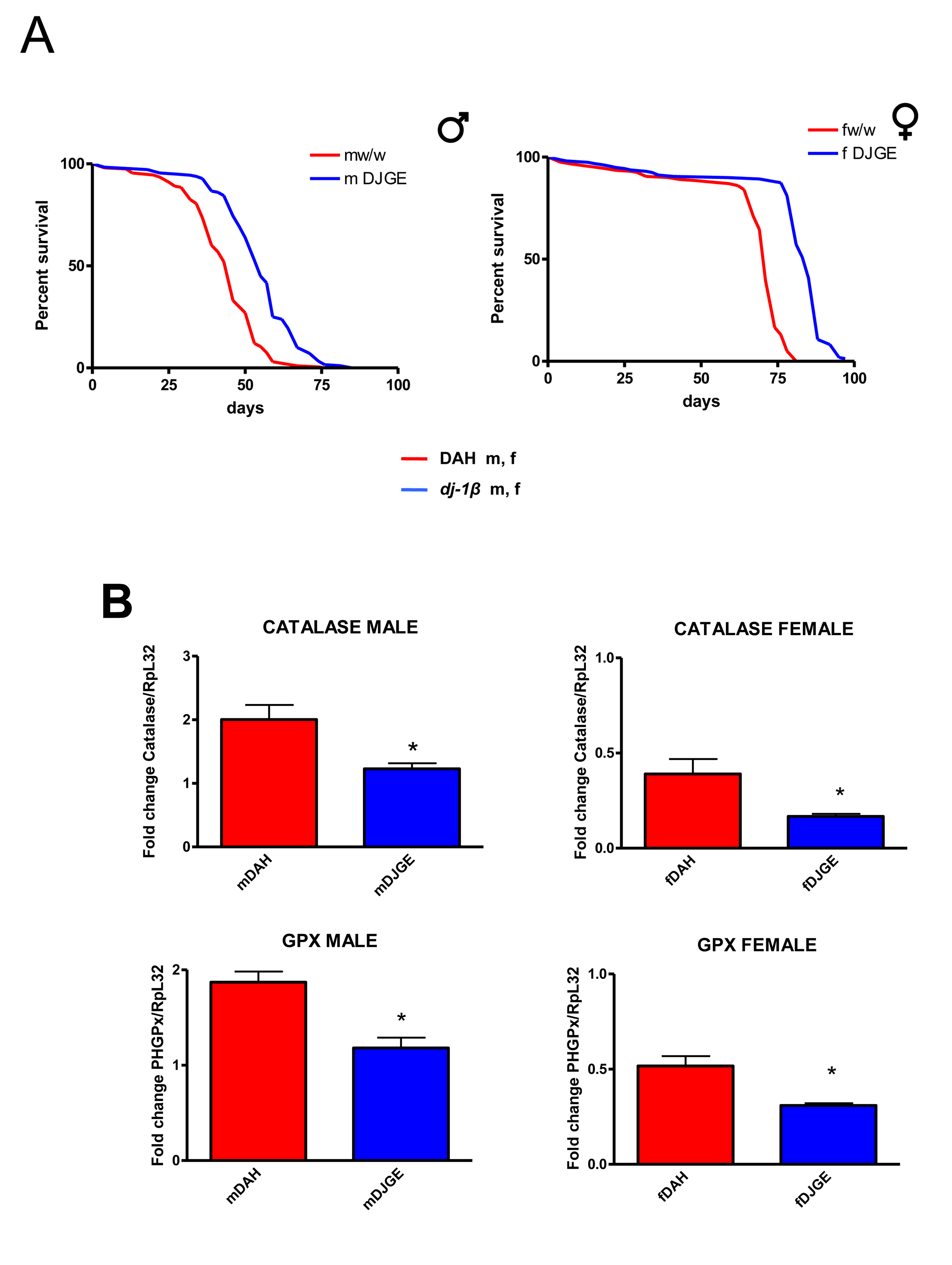

Supplement: Supplementary Figure 7 — (A) Survival curves of dj-1β mutant flies backcrossed during seven generation in dahomey background. Combined data from two independent experiments using 100 flies per group per experiment Mean, maximum lifespans (d) were: DAH males (46, 55); DAH females (71, 76); dj-1β mutant males (55, 64); dj-1β mutant females (85, 88). (B) The level of expression of four antioxidant genes: catalase (CAT), glutathione peroxidase (PHGPx), superoxide dismutase 1 (SOD1) and superoxide dismutase 2 (SOD2) were analyzed by qPCR. Plotted data are mean + SEM. * denotes statistically significant differences between groups (T-test, p<0.05, n =5-6 samples per group). [file aging-02-200-s007.tif]

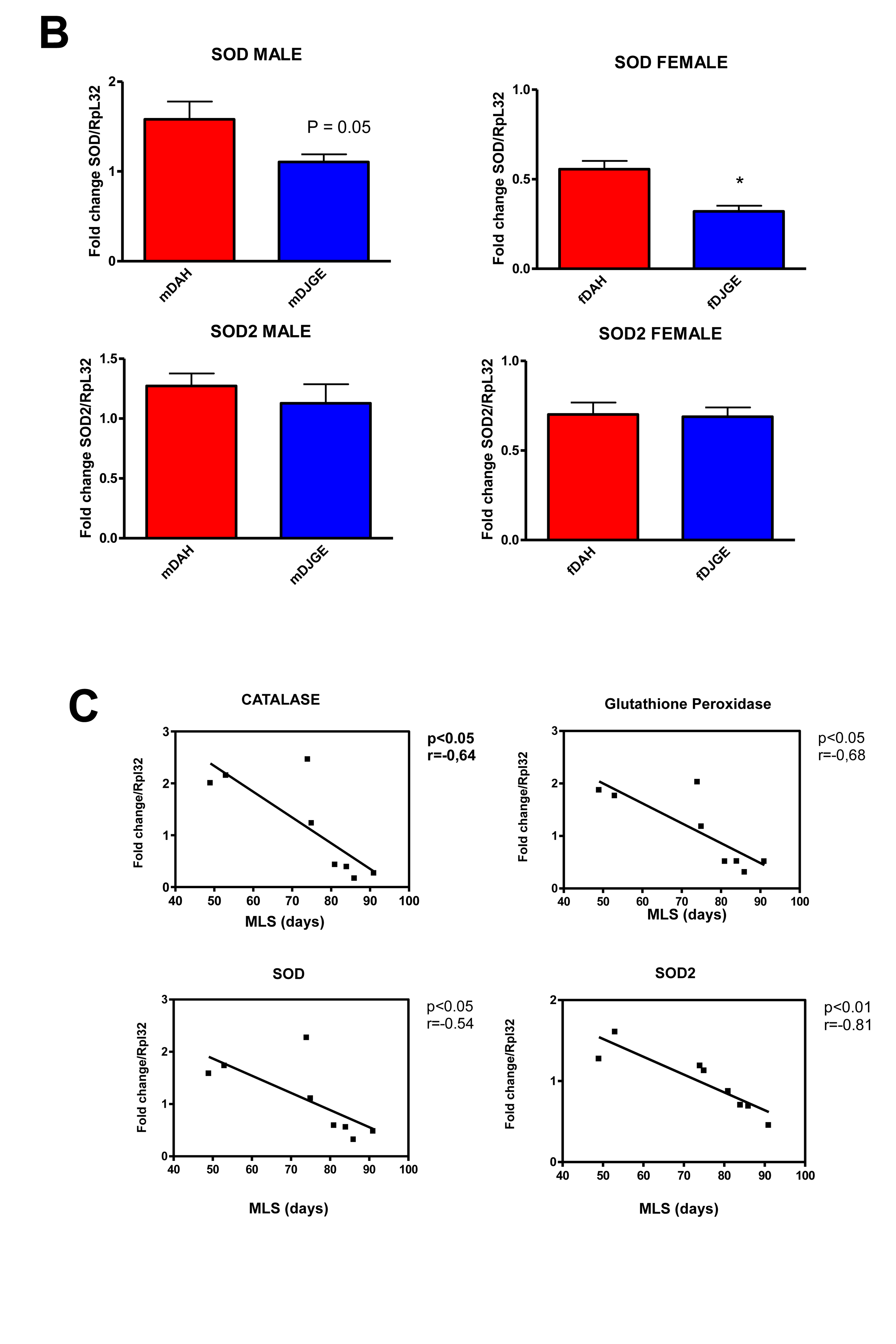

Supplement: Supplementary Figure 8 [file aging-02-200-s008.tif]
