# Supplementary material for: Mitochondrial ROS production correlates with, but does not directly regulate lifespan in drosophila
Source: Aging (Albany NY). 2010 Apr 15;2(4):200–23. doi: 10.18632/aging.100137 (PMC2880708; doi:10.18632/aging.100137)
Supplement: Supplementary Table 1 — AOX transgenic flies were from line F24 and were 2-3 d old. Results are mean ± SEM. Number of independent samples in parentheses [file aging-02-200-s001.doc]

**Supplementary Table 1. Mitochondrial oxygen consumption (nmol O_2_/min.mg prot) in AOX-expressing (AOX/da-GAL4) and non-expressing (AOX/–) transgenic flies**

|  | **AOX/–** | | **AOX/da-GAL4** | | **ANOVA** |
| --- | --- | --- | --- | --- | --- |
|  | Pyruvate + Proline | | | | |
| State 4 | 27 ± 2 (4) | 23 ± 2 (4) | 34 ± 5 (5) | 38 ± 4 (6) | NS |
| State 3 | 378 ± 37 (4) | 379 ± 40 (4) | 418 ± 42 (5) | 459 ± 47 (6) | NS |
| RCI | 16 ± 1 (4) | 18 ± 3 (4) | 18 ± 3 (5) | 14 ± 1 (6) | NS |
|  | sn-glycerol-3-Phosphate + rotenone | | | | |
| State 4 | 97 ± 18 (5) | 99 ± 5 (5) | 145 ± 9 (5) | 119 ± 12 (5) | NS |
| State 3 | 268 ± 33 (5) | 263 ± 31 (5) | 266 ± 24 (5) | 259 ± 37 (5) | NS |
| RCI | 3 ± 0.6 (5) | 2.7 ± 0.3 (5) | 1.7 ± 0.2(5) | 2.2 ± 0.4 (5) | NS |

AOX transgenic flies were from line F24 and were 2-3 d old. Results are mean ± SEM. Number of independent samples in parentheses
