# Supplementary material for: Mitochondrial ROS production correlates with, but does not directly regulate lifespan in drosophila
Source: Aging (Albany NY). 2010 Apr 15;2(4):200–23. doi: 10.18632/aging.100137 (PMC2880708; doi:10.18632/aging.100137)
Supplement: Supplementary Table 2 [file aging-02-200-s002.doc]

**Supplementary Table 2. Primer sequences used for qPCR and sequencing**

| **Primer** | **Sequence** |
| --- | --- |
| RpL32-f | GTTCGATCCGTAACCGATGTT |
| RpL32-r | CACCAGTCGGATCGATATGC |
| Cat1 | TGATTCCTGTGGGCAAAATG |
| Cat2 | CAGACGACCATGCAGCATCT |
| Sod1 | GAACTCGTGCACGTGGAATC |
| Sod2 | GGTGGTTAAAGCTGTCTGCGTA |
| SodII1 | GTCTGGTGGTGCTTCTGGTG |
| SodII2 | GCCCGTAAAATTTCGCAAAC |
| PHGPxF | AGGTGTTCGCCAAGGTAAGAC |
| PHGPxR | GGTCTGCTTGGCCTTTAGGTA |
| CoIF2 | GGAGGATTACCTCCATTTTTAGG |
| CoIR4 | CTCCTGTTAATCCTCCTACTG |
| CoIF4 | CCTGGAGCATTAATTGGAGATG |
| CoIR5 | CTCCTAAAGCAGGTACTGTTC |
