# Supplementary material for: Mitochondrial ROS production correlates with, but does not directly regulate lifespan in drosophila
Source: Aging (Albany NY). 2010 Apr 15;2(4):200–23. doi: 10.18632/aging.100137 (PMC2880708; doi:10.18632/aging.100137)
Supplement: Supplementary Table 3 [file aging-02-200-s003.doc]

**Supplementary Table 3. Nucleotide variation at polymorphic sites of the cytochrome c oxidase I (CoI) gene in three wild type strains of *Drosophila melanogaste***

|  | mtDNA | | |
| --- | --- | --- | --- |
| Polymorphic site | Oregon R | Canton S | Dahomey |
| 1 (1512) | T | C | T |
| 2 (1674) | A | A | G |
| 3 (1779) | T | T | A |
| 4 (1836) | G | T/G | A |
| 5 (1861) | C | C | T |
| 6 (1929) | A | A | G |
| 7 (2160) | C | T | C |
| 8 (2186) | T | T | C |
| 9 (2863) | T | T | C/T |
| 10 (2964) | A | A | G |
